# Pain during Cast Wedging of Forearm Shaft and Distal Forearm Fractures in Children Aged 3 to 12 Years—A Prospective, Observational Study

**DOI:** 10.3390/children7110229

**Published:** 2020-11-16

**Authors:** Florian Freislederer, Tobias Berberich, Thomas O. Erb, Johannes Mayr

**Affiliations:** 1 Department of Pediatric Surgery, University Children’s Hospital Basel (UKBB), 4031 Basel, Switzerland; johannes.mayr@ukbb.ch; 2 Department of Pediatric Surgery, St. Elisabethen Hospital, 79539 Lörrach, Germany; t.berberich@elikh.de; 3 Department of Pediatric Anesthesia, University Children’s Hospital Basel (UKBB), 4056 Basel, Switzerland; Thomas.Erb@ukbb.ch

**Keywords:** cast, child, conservative, fracture, pain, radius, visual analog scale, cast wedging

## Abstract

Background: Although fracture displacement in children is easily treated by cast wedging, no data on pain associated with the procedure are available. We hypothesized that there is no clinically relevant difference in pain before and after cast wedging in children between 3 and 12 years of age. Patients and Methods: This international, multicenter, prospective, observational study included 68 children (39 male, 29 female) aged 3 to 12 years (median age 8 years) with forearm fractures. Cast wedging was performed 5 to 10 days after the injury. Before starting the procedure, we administered a single oral dose of sodium metamizole (10 mg/kg body weight), and the children inhaled a nitrous oxide/oxygen mixture (50%/50%) during the wedging procedure. Pain was rated on a visual analog scale (VAS) 5 to 10 min before incision of the cast as well as 3 to 5 min and 30 min (maximum remembered pain) after inhalation stop. The degree of bending was judged either by the surgeon or was determined on the basis of first signs of pain expressed by the patient. We assessed the effectiveness of the procedure by obtaining X-ray images in two planes after 3 to 9 days. Results: Among the 68 patients, median VAS score before cast wedging was 0. This increased to a score of 1 (*p* = 0.015) at 3 to 5 min after the procedure. Median VAS score for the maximum remembered pain measured after 30 min was 0. Median differences in angulation between proximal and distal bone fragments before and after the intervention were 0° (*p* < 0.0001) in the a.p. view and 8.4° (*p* < 0.0001) in the lateral view. Conclusion: Cast wedging improved the position of forearm fracture fragments at the expense of minimal short-term pain.

## 1. Introduction

One third of all pediatric fractures involve the forearm [1,2,3,4]. Up to the age of 12 years, most closed forearm fractures can be treated nonoperatively with a cast if rotational malalignment, neurovascular impairment, or compartment syndrome are absent. The growing skeleton differs from the adult skeleton by having a correction mechanism for fracture-induced displacement [5]. Corrections of axial deviations in the frontal and sagittal plane occur in the periosteal-endosteal and physeal regions of bones. These corrections are especially effective in the area of the proximal humerus and distal forearm. Side-to-side displacements up to the width of the shaft are corrected by periosteal remodeling until the age of 10 to 12 years. However, the proximal end of the radius is not able to adequately correct side-to-side displacements. Furthermore, spontaneous correction of rotational malalignment is proven only for fractures of the humerus and femur in young children [1,6].

In principle, any axial deviation in the frontal and sagittal plane of the forearm or lower leg can be reduced by cast wedging, so that the remaining angulation resides within the limits of spontaneous correction [1,2,3,7,8,9,10]. The wedging procedure should be performed between the 6th and 10th day after the injury by cutting the cast, bending it to regain better orientation of the two parts of the fractured bone, and inserting a placeholder into the gap to stabilize the angulation of the cast and position of the fragments [7,8]. Conducting the wedging procedure 6 to 10 days after the injury allows tissue swelling to subside and helps to minimize pain related to the correction of bone malalignment [1]. Usually, this pain subsides within a few minutes. Although current wedging therapy is accompanied by the use of nonopioid analgesics, minimal pain still occurs during the procedure and is interpreted as a sign of treatment success [1,11]. On the other hand, any pediatric treatment should be done as painlessly as possible [12]. Thus, these two principles may contradict each other, which has led to controversial discussion of the wedging therapy.

Fracture treatment under general anesthesia is an alternative technique to reduce residual malalignment of long-bone fractures. However, treatment of these frequent fractures under general anesthesia is questionable because it is associated with several risks, especially in children [13]. To the best of our knowledge, neither pain measurements during cast wedging therapy nor any data on the effectiveness of the accompanying analgesic therapy are available in the literature. Therefore, the aim of the current study was to prospectively assess pain scores obtained at several time points during forearm cast wedging, assuming that pain therapy during cast wedging is effective and complies with current pain treatment guidelines [11].

## 2. Patients and Methods

This international, multicenter, prospective, observational study included 68 children (39 male, 29 female) aged 3 to 12 years (median age 8 years, IQR age 4 years) with forearm fractures. The study participants were recruited from outpatients who were followed up after primary conservative treatment of forearm fractures immobilized with a cast.

First, we aimed to examine whether there is a clinically relevant difference in pain perception before forearm cast wedging compared to pain perceived 3 to 5 min after forearm cast wedging in children aged 3 to 12 years. Second, we recorded the maximum pain during cast wedging as remembered by the child (maximum remembered pain) 30 min after the wedging procedure. We also compared the radiologic angles measured between the proximal and distal bone fragments before and after cast wedging to determine treatment success.

In line with usual procedures, the indication for cast wedging was established by a senior pediatric surgeon or pediatric orthopedic surgeon after obtaining a.p. and lateral radiographs 6 to 10 days after the injury.

### 2.1. Inclusion Criteria

We included children who suffered fractures with rotational malalignment, shortening, and fracture instability requiring operative stabilization. Patients aged 3 to 12 years were selected for the cast wedging procedure by a senior pediatric or orthopedic surgeon. Written informed consent was obtained from parents or caregivers before entering the child into the study. We included only children in good physical condition according to classification I or II defined by the American Society of Anesthesiologists [14].

### 2.2. Exclusion Criteria

Children with intolerance to nitrous oxide or metamizole sodium and/or pre-existing neuromuscular, rheumatic, or musculoskeletal disease were excluded from this study.

### 2.3. Study Procedures

The study, which was approved by the local Ethics Committee (protocol number: BASEC 2017-02247), was performed at the University Children’s Hospital Basel (UKBB), Switzerland, and the communal Hospital St. Elisabethen, Lörrach, Germany. These centers were selected because the two departments of pediatric surgery employ identical treatment strategies for forearm fractures and have been entertaining a well-established cooperation in clinical and scientific activities. We hypothesized that there is no clinically relevant difference in pain perception before and after cast wedging.

The primary study endpoint was the maximum pain experienced 5 min before (PainBE) and 5 min after (PainAF) cast wedging to assess the influence of cast wedging on pain perception. Pain was recorded with a validated visual analog scale (VAS [15]), where the child specifies the current pain by pointing on one of 6 different faces with the use of a card slider. The back of the card indicates the corresponding numerical value ranging from 0 to 10. If the child pointed to a value in the middle between two possible values, the next lower value was documented. This technique represents a standard procedure at both study centers to assess perioperative pain in children. Patient characteristics (age, gender, body weight) were recorded without expecting any influence on the primary endpoint.

**Figure 1:**
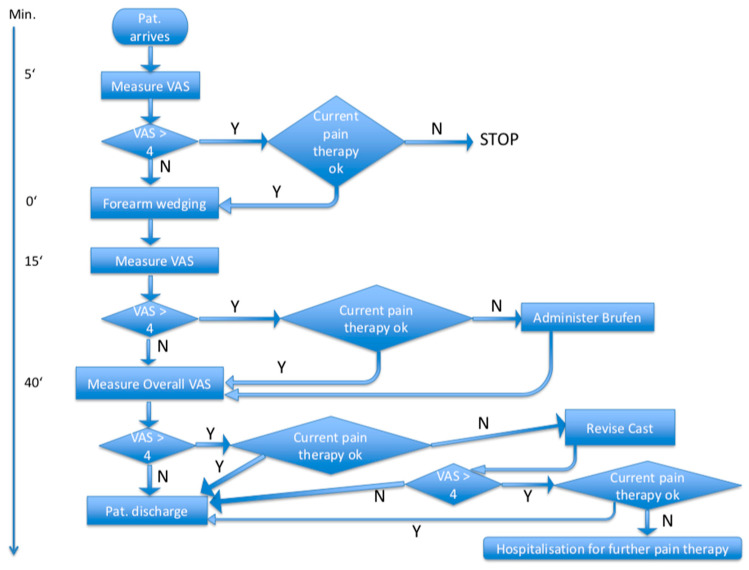
Flowchart of timetable and pain management (N: no, Y: yes). VAS: visual analog scale.

Secondary endpoints of the study included the assessment of maximum remembered pain during the procedure (PainOv) as elicited at 30 min after the intervention. This time interval was selected because some children reported drowsiness shortly after stopping inhalation of the nitrous oxide/oxygen mixture. In addition, angle differences in a.p. and lateral views between the proximal and distal bone fragments before and 3 to 9 days after the wedging procedure were assessed as the secondary study endpoint. Angle differences in a.p. and lateral views before (Angap/lat_1) and 3 to 9 days after (Angap/lat_2) the wedging procedure were measured electronically by two independent, nonblinded radiologists at each study center. To obtain comparable radiographic views, X-ray imaging was standardized by drawing a cross on the cast in line with the laser cross of the radiographic device to ensure consistent rotational position of the forearm and cast. Blinding was not possible because of the radiologically visible gap in the cast. For ethical considerations, necessary actions were taken after each pain measurement, depending on the step within the study (Figure 1).

**Figure 2:**
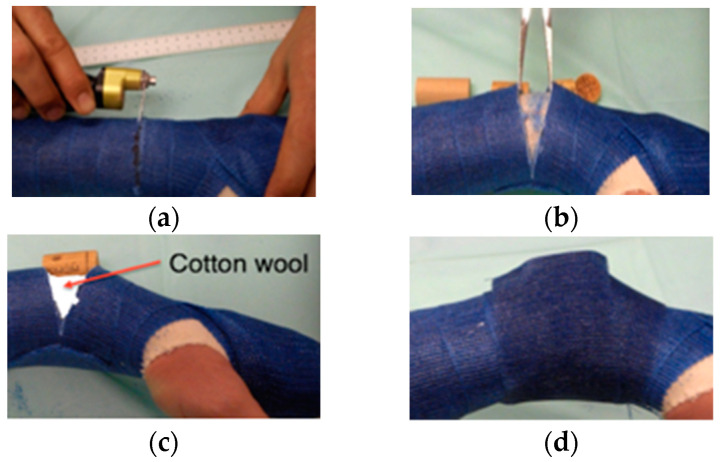
Cast wedging of a distal forearm fracture. (**a**) Wedging position is marked, and the cast is cut approximately 2/3 of the circumference using an oscillating saw. (**b**) Cast can now be opened with a clamp until the desired correction is obtained. (**c**) Position is fixed using a placeholder (i.e., piece of cork), and cotton wool is inserted at the wedging site to serve as a cushion. (**d**) Cast is stabilized with additional circular layers of synthetic cast.

Approximately 30 to 60 min before starting the wedging procedure, we administered a single oral dose of metamizole sodium (10 mg/kg body weight). Split casts were closed before the wedging procedure. The wedging line on the cast was defined by the senior attending physician by clinical judgment (for more distal fractures, the wedging line marked on the cast was placed more proximally [1]). Before cutting the cast, care was taken not to place the line of wedging at the fracture site. This facilitated the subsequent distinction between pain occurring at the fracture site and pain caused by kinking of the angulated cast at the line of wedging. We augmented mixed soft/hard plaster casts at the region of the intended cast wedging line by applying 2 to 3 circular layers of hard cast. The child was asked to inhale a 1:1 mixture of nitrous oxide/oxygen by face mask for at least 2 min prior to the procedure until the end of the procedure. In line with these standard procedures, the cast was cut (approximately 2/3 cast circumference) in a direction perpendicular to the longitudinal axis of the forearm at the lowest point of concavity of the axial deviation. The cast was bent manually or with a clamp inserted into the gap according to the judgment of the attending surgeon or until the child started to complain of minimal pain due to compression of the convex side of the fracture. The new position was fixed by inserting a placeholder (radiolucent piece of cork or wood) into the gap in the cast. Subsequently, we inserted soft padding into the remaining gap in the cast. We waited several minutes to observe whether the child tolerated the angulation of the cast. Finally, we stabilized the angulated area of the cast with two additional circular layers of cast (Figure 2).

### 2.4. Statistical Analyses

To assess the difference between pain before and after the wedging procedure (primary endpoint), the data were analyzed as a noninferiority trial. The difference between perceived pain after and before wedging was estimated using paired analysis and 95%, confidence intervals (CIs) were calculated. Noninferiority was concluded if the 95% CI did not cross the predetermined noninferiority margin (δ). A clinically relevant noninferiority margin was defined as 1 VAS unit. For meaningful clinical use, the measured number was rounded to the next lower integral number.

Our study hypothesis H0 was as follows: Maximum pain 3 to 5 min after wedging (PainAF) is inferior (higher or equal) by at least δ compared to maximum pain before wedging (PainBE), where δ is the clinically relevant noninferiority margin.

Data from pilot measurements were used to calculate the sample size. We simulated data for each sample size based on multivariate truncated distributions (truncated to lie between 0 and 10) and analyzed the data according to the primary analysis described below. For each sample size, simulation and analysis were performed 1000 times; the proportion of cases in which noninferiority was shown was taken as the power of the study with the relevant sample size. Sample sizes from 30 to 90 were simulated. With a sample size of 64 participants, the simulation showed an 80% power to determine noninferiority of pain after wedging compared to pain before wedging with a difference margin (δ) of 1 point and significance level of 0.05.

Nonadherence to the protocol was expected to be low. Because very few variables were measured and a single visit was necessary during the study, loss of data was expected to be negligible. Therefore, the difference between the intention-to-treat and per-protocol analyses was not taken into account. For missing values, the reasons were recorded.

Procedural data were analyzed for normal distribution using the Shapiro–Wilk test. To test for noninferiority of pain after wedging compared to pain before wedging, the differences of pain scores after and before wedging therapy were compared to the noninferiority margin by a paired *t*-test using JMP^©^ Software (Version 12, SAS, Cary, NC, USA). The following parameters were recorded and described by summary statistics: age, sex, body height, body weight, pain before wedging, pain 3 to 5 min after stopping inhalation of nitrous oxide/oxygen mixture, maximum pain perception during the whole procedure (secondary endpoint), and angle of the radius fracture before and after wedging of the cast (secondary endpoint). A *p*-value of ≤0.05 was considered significant.

## 3. Results

From June 2018 to April 2020, we enrolled 68 children (39 males, 29 females) with forearm fractures of various types (Table 1).

One additional patient with complete distal diaphyseal radius fracture was excluded because a painful cast had to be removed one day after cast wedging, and this patient could not be followed up. After removal of the cast, the pain subsided, and there were no other complications in this patient.

**Figure 3:**
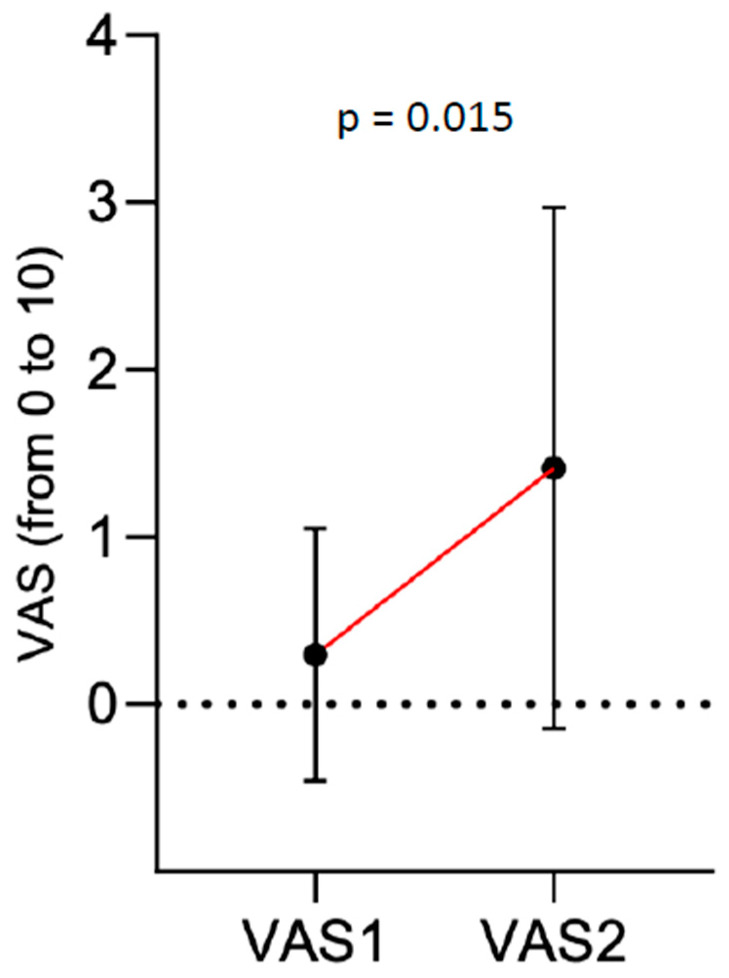
Difference of median visual analog scale (VAS) scores 1 and 2 (VAS 1: 5 min before wedging, VAS 2: 3–5 min after wedging).

Median VAS score 5 min before cast wedging was 0 (IQR 0). This increased to a VAS score of 1 at 3 to 5 min after stopping the inhalation of nitrous oxide/oxygen mixture (IQR 2). This difference was significant (*p* = 0.015) (Figure 3).

The median VAS score for the maximum pain remembered 30 min after stopping inhalation of nitrous oxide/oxygen mixture was 0 (IQR 1).

**Figure 4:**
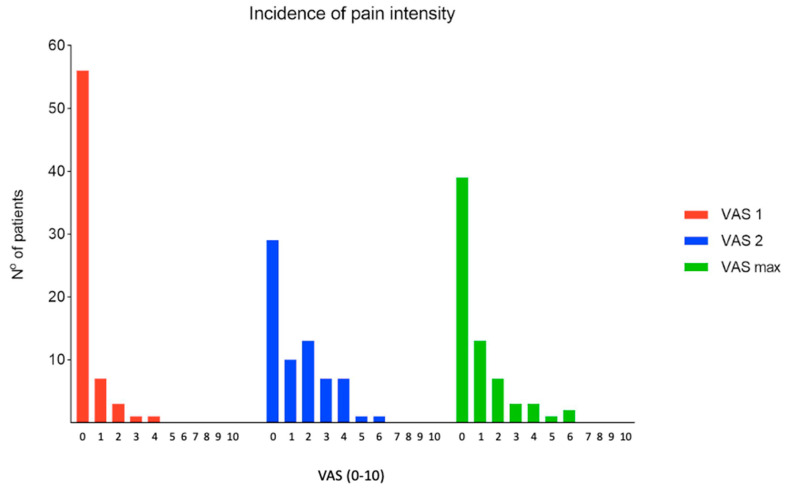
Pain distribution at each time point: VAS1: 5 min before cast wedging, VAS2: 5 min after cast wedging, VAS max: maximum pain memorable 30 min after cast wedging.

One (1.5%) patient achieved a VAS score >4 (i.e., score of 6) 3–5 min after stopping the inhalation of nitrous oxide/oxygen mixture. The pain subsided to below 4 after oral administration of ibuprofen. Another 2 (2.9%) patients reported a VAS score of 6 for maximum remembered pain as elicited 30 min after stopping nitrous oxide/oxygen inhalation, but these patients did not have a VAS score >4 measured 3–5 min after the wedging procedure (Figure 4).

**Figure 5:**
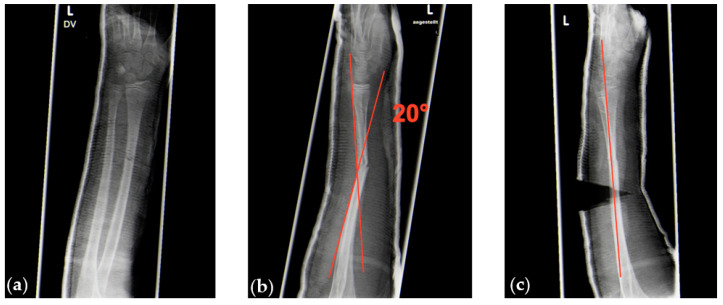
(**a**,**b**) Radiologic images of a sagittal 20° angular deformity of a greenstick fracture between midshaft and distal forearm shaft region without deformity in the coronal plane (**a**). (**c**) Complete correction of the deformity after cast wedging.

Among patients with marked displacement of bone fragments, 5 of 15 (33.3%) patients with overall angular correction of >15° reported a VAS score of ≥4 measured 3–5 min after stopping nitrous oxide/oxygen inhalation (Figure 5).

Maximum remembered pain recorded 30 min after stopping the inhalation dropped to ≤2 in 12/15 (80%) patients. The remaining three patients reported a VAS score of ≤4 at 30 min after stopping nitrous oxide/oxygen inhalation.

**Figure 6:**
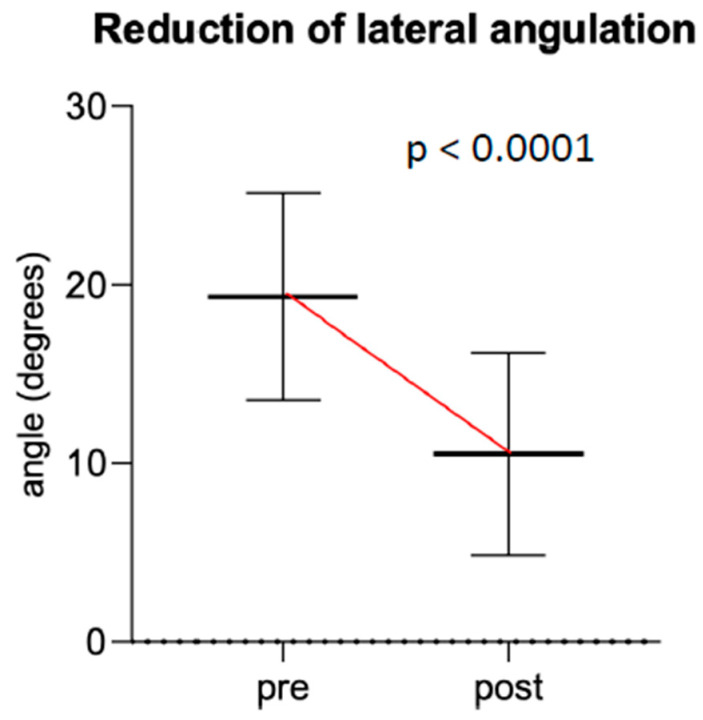
Change in median sagittal angulation before and after cast wedging.

**Figure 7:**
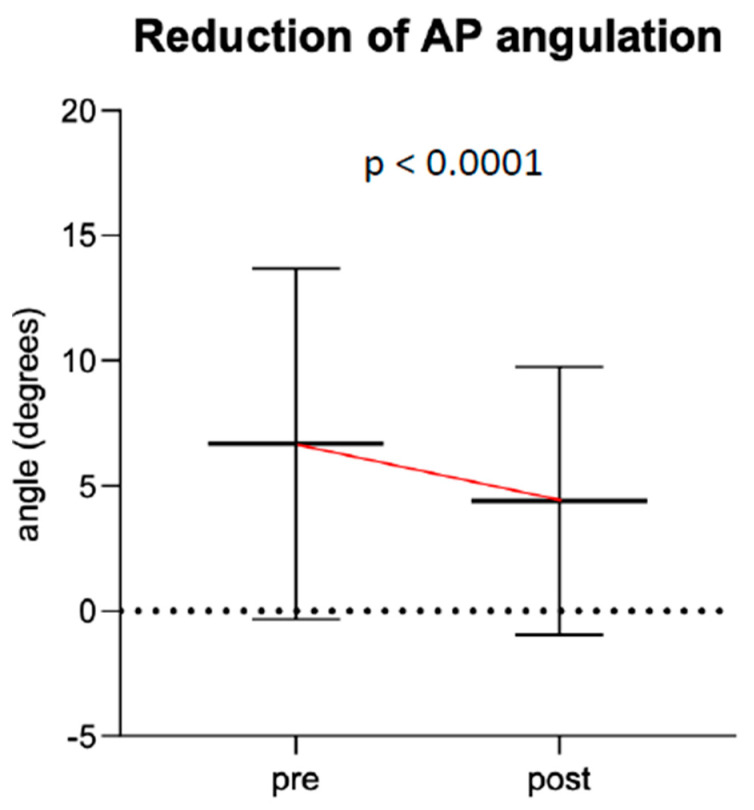
Change in median coronal angulation before and after cast wedging.

We grouped our patients into a younger “preschool” group (aged 3–7 years) and older “school age” group (aged 8–12 years). Both groups experienced a change between the VAS scores before and 3 to 5 min after the wedging procedure, although the change was significant only in the school age group (*p* = 0.023 vs. *p* = 0.200 in the preschool group). In school age children, the initial median VAS score of 0 increased to 2 at 3 to 5 min after cast wedging (*p* = 0.023). Median fracture angulation in these two groups improved by 8.4° on lateral view and 0° on a.p. view (IQR −7° (lat) and −3.9° (a.p.)) (Figure 6 and Figure 7). Regarding the fracture type, there was no statistically significant difference in angulation improvement between type 1 (metaphyseal) and type 3 (shaft) fractures (*p* = 0.67).

## 4. Discussion

Despite the current trend towards surgical treatment of forearm fractures in children living in developed countries [3], cast wedging represents a promising noninvasive method to correct angular bone deformity of long-bone fractures in the pediatric population [1,8,10,16]. This procedure enables gentle, closed reduction thus avoiding the risks of sedation or surgical intervention while minimizing treatment costs [17]. Our study proved cast wedging to correct the alignment of fracture fragments in pediatric distal and shaft fractures of the forearm with axial deviation at the expense of transient, minimal pain. We investigated this clinically relevant question exclusively in children aged 3 to 12 years because of the age-specific bone growth and anatomy, as well as the frequency of forearm fractures in this age group.

Cast wedging was first reported in the first half of the 20th century, but studies on cast wedging are sparse. A meta-analysis identified only three studies including a total of 325 patients [16]. According to these studies, cast wedging is a safe alternative to surgical correction of forearm fractures whose fracture position was deemed unacceptable after one week. Cast wedging in forearm fractures proved unsuccessful in 12/262 (4.5%) patients. Only one of these studies investigated cast wedging in forearm fractures in a prospective manner [10]. The authors opted for cast wedging in the presence of fracture displacement by more than 20° in any place for distal radius fractures and >15° in any plane for fractures of the middle third shaft of the forearm. Combining literature data on forearm fracture patterns and experts’ opinions, Ploegmakers introduced limits of acceptable angular deformities for different types of pediatric forearm fractures but did not separately evaluate sagittal and coronal deformities [18]. Von Laer defined the limit for spontaneous correction as a 10° axial deviation in children aged 6 to 12 years for proximal forearm shaft fractures, 20° for midshaft greenstick fractures and complete fractures in children aged 3 to 5 years, and 10° in children aged 6 to 12 years [1]. For distal forearm fractures, von Laer set a limit of 10° in the frontal and 30° in the sagittal plane [1].

**Figure 8:**
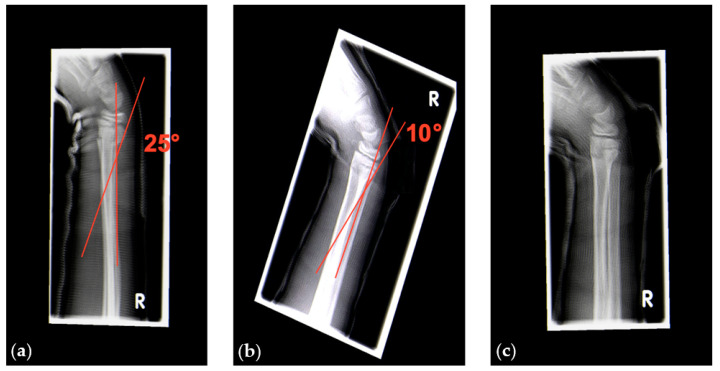
(**a**) Radiographic images of a sagittal 25° angular deformity of a dorsally displaced complete fracture of the distal radial metaphysis. (**b**,**c**) Angulation after cast wedging reduced by 15°, moving the deformity into the limits of sufficient spontaneous correction.

We performed radiographic controls 5 to 10 days after trauma and decided whether to perform cast wedging or not. Our indications for cast wedging followed the guidelines published by von Laer [1]. Furthermore, we decided to perform cast wedging also for angular deformities >20° in the sagittal plane in distal forearm fractures to prevent further displacement (Figure 8).

We did not notice any angle correction failures after wedging. However, the cast of one patient had to be replaced one day after the wedging procedure due to persisting pain.

Samora et al. investigated 61 cast wedging procedures in forearm fractures but applied cast wedging up to the age of 16 years for males and 14 years for females [10]. They observed similarly favorable success rates for cast wedging. One 14-year old male patient experienced residual angular deformity after cast wedging and underwent surgical fixation. Three of their patients experienced pain in the evening after cast wedging, and two of these patients had pain for up to 2 days after the cast wedging procedure. This pain was described as mild and responded to over-the-counter (OTC) analgesics, but “mild” pain was not further described or measured [10]. In our study, we achieved a median correction of total angular deformity of 10°. In 7/68 (10%) patients, only minimal correction (≤3°) was achieved.

Immobilization for fracture healing in children is not a static process, and changes in alignment can occur over time once tissue swelling decreases, especially if the position of immobilization is not corrected or has been modified. Therefore, even a small correction in alignment during the first 2 weeks after injury can be the decisive factor for fracture healing in acceptable alignment of the bone fragments [9].

## 5. Study Limitations and Strengths

Our study had a number of limitations. First, we did not consistently document the wedging site and were thus unable to correlate wedge position with outcome. The wedging procedure used was based on previous experience gained in a pilot study [19]. The pilot study aimed to develop a forearm bone fracture model and to systematically evaluate biomechanical characteristics. The data indicated how cast materials, wedge location, and wrist position affected the efficiency of wedging to correct the deformity [19].

Second, we did not assess long-term function of the upper extremity of the children in our study. Thus, we cannot prove that the wedging therapy was necessary in all cases, although we achieved good radiologic outcomes. We successfully shifted most fracture displacements into the range of angulations known to permit spontaneous correction of the deformity. Nonetheless, we were not able to answer the question whether the seven patients in whom only minimal angular correction (≤3°) was achieved would have had a similarly favorable outcome in the absence of cast wedging.

Third, due to ethical concerns we were not able to include a control group to prove the superiority of our therapy over cast therapy alone or surgical treatment.

Finally, in the absence of a control group, it remains uncertain whether inhaling nitrous oxide/oxygen mixture (50%/50%) was the decisive factor for reducing pain during cast wedging.

The strengths of this study include the use of a standardized wedging procedure at both centers and involvement of a small specialist team. Cast wedging therapy is a standardized and well-defined procedure and is part of current pediatric surgical training. Use of the pain VAS according to Hicks [15] is established in everyday clinical practice and is routinely used to evaluate and record pain in children at both study centers. We minimized bias by using this validated pain VAS for children and by marking the casts for the laser-targeting device of X-ray apparatus. This ensured consistent X-ray projection at follow-up at the two centers.

## 6. Conclusions

We showed that cast wedging in children improved the alignment of fracture fragments in distal metaphyseal and diaphyseal forearm fractures with axial deviation at the expense of minimal transient pain. Although we were able to show a significant difference in pain before and after treatment according to our hypothesis, the actual increase from a median VAS score of 0 before to a median VAS score of 1 after treatment was minimal. Moreover, maximum pain remembered by the children at 30 min after the intervention showed no relevant increase of pain compared to the pain they perceived before the intervention.

In conclusion, we consider cast wedging an effective and child-friendly therapy of bone fragment misalignment in radius and forearm fractures requiring reduction or prevention of further displacement in the first 10 days after trauma.

## Figures and Tables

**Table 1:** Fracture types in the study population (*n* = 68).

Type of Fracture			Salter Harris Type I (Epiphyseal Separation)	Greenstick	Bowing	Complete	Multi-Fragment	Buckle Fracture
Type 1	Metaphyseal forearm	Radius and ulna	1	5	0	4	0	0
		Radius		3	2	7	0	2
		Ulna		0	0	0	0	0
Type 2	Distal diaphyseal forearm	Radius and ulna		6	0	9	0	1
		Radius		6	2	6	0	0
		Ulna		0	0	0	0	0
Type 3	Forearm shaft	Radius and ulna		7	0	2	0	0
		Radius		4	0	1	0	0
		Ulna		0	0	0	0	0

## Abbreviations

a.p.: antero-posterior; CI: confidence interval(s); NSAIDs: nonsteroidal anti-inflammatory drugs; VAS: visual analog scale
